# 

**Published:** 1879-06

**Authors:** 


### TABLE OF CONTENTS.

### ORIGINAL COMMUNICATIONS.

RECENT DERMATOLOGICAL LITERATURE UPON "NEW FORMATIONS." *By Edward Wigglesworth, M. D., Boston, Mass.* .......... 129
TREATMENT OF BURNS AND ERYSIPELAS BY WHITE LEAD. *By S. G. Riley, M. D., Shiloh, Ga* .......... 139
AN INTERESTING CASE OF STRICTURE OF FEMALE URETHRA AND TREATMENT. *By H. L. Wilson, M. D., Atlanta, Ga* .......... 142

### REPORTS OF SOCIETIES.

Atlanta Academy of Medicine .......... 144
New York Academy of Medicine .......... 145
Cincinnati Medical Society .......... 152

### SELECTIONS.

A Case of Fistula in Ano, with Remarks .......... 161
Psoriasis .......... 165
Veratrum Viride in Puerperal Eclampsia .......... 166
A Case of Meningeal Hemorrhage .......... 169
A Case of Lithotomy .......... 174
Painless Method of Excising the Whole Tongue .......... 176

### EDITORIAL.

Medical Education .......... 180

The State Medical Association .......... 183

Meteorological Report for May .......... 184

### BIBLIOGRAPHICAL.

A Clinical Treatise on Diseases of the Liver .......... 185

A Treatise on Therapeutics .......... 185

Spermatorrœa .......... 186

Pamphlets Received .......... 187

### EXCERPTA.

Hahnemann and Homœopathy .......... 188

